# SMS text pre-notification and delivery of reminder e-mails to increase response rates to postal questionnaires: a factorial design, randomised, controlled trial

**DOI:** 10.1186/1745-6215-16-S2-P88

**Published:** 2015-11-16

**Authors:** Kath Starr, Gladys McPherson, Mark Forrest, Seonaidh Cotton

**Affiliations:** 1 University of Aberdeen, Aberdeen, UK

## Background

Participant non-response to postal questionnaires in randomised controlled trials (RCT) can jeopardise trial results.

## Methods

We evaluated two interventions aimed at increasing postal questionnaire response rates in a 2x2 partial factorial RCT nested within a single, large, UK-wide RCT of medical expulsive therapy for ureteric stones. The interventions were: questionnaire pre-notification via SMS text prior to posting the trial questionnaires versus no pre-notification; for non-responders to the initial questionnaire mailing, use of an e-mail reminder (containing a hyperlink for online questionnaire completion) versus a postal reminder with paper questionnaire. Participants were randomised to the pre-notification comparison, the reminder comparison or both, depending on whether they supplied mobile telephone number and/or email address. The primary outcome was response to 4 and 12 week questionnaires.

## Results

418 participants were randomised to the SMS pre-notification comparison: the intervention had no effect on response rates at either questionnaire time point. In sub-group analyses, SMS pre-notification increased response rates in women (p=0.038), but only at the first time-point. 119 participants were randomised to the reminder comparison. There was no difference in response rates between those randomised to e-mail or postal reminders.

## Conclusion

SMS text pre-notification of questionnaire delivery and email delivery of reminders did not improve response rates. There was some evidence to suggest SMS text pre-notification may be effective in women. While e-mail reminders had no impact on response rates, their use could result in postage cost savings. Further studies of these interventions, nested in different RCTS, are warranted, with subsequent meta-analysis.

